# Multimodality imaging features of desmoid tumors: a head-to-toe spectrum

**DOI:** 10.1186/s13244-020-00908-0

**Published:** 2020-09-25

**Authors:** F. Rosa, C. Martinetti, F. Piscopo, D. Buccicardi, D. Schettini, C. E. Neumaier, N. Gandolfo, L. Grazioli, A. Gastaldo

**Affiliations:** 1 Diagnostic Imaging Department, San Paolo Hospital-ASL 2, via Genova, 30 Savona, Italy; 2 Department of Health Sciences (DISSAL), University of Genova, via A. Pastore 1, 16132 Genova, Italy; 3 Diagnostic Imaging Department, Villa Scassi Hospital-ASL 3, Corso Scassi 1, Genova, Italy; 4 Diagnostic Imaging and Senology Unit, Policlinico San Martino, Largo R. Benzi 10, 16132 Genova, Italy; 5 ASST “Spedali Civili”, P.le Spedali Civili 1, 25123 Brescia, Italy

**Keywords:** Desmoid tumors, Computed tomography, Ultrasound, Magnetic resonance

## Abstract

Desmoid tumors (DTs) are a rare and biologically heterogeneous group of locally aggressive fibroblastic neoplasm: their biological behavior spectrum ranges from indolent to aggressive tumors. DTs are classified as intra-abdominal, extra-abdominal, and within the abdominal wall lesions.

It is well known that abdominal and extra-abdominal DTs are associated with familial adenomatous polyposis (FAP) and Gardner syndrome. Possible risk factors are prior trauma/surgery, pregnancy, and oral contraceptives.

There was a real revolution in the management of DT: from aggressive first-line approach (surgery and radiation therapy) to a more conservative one (systemic treatment and “wait-and-see policy”).

In these clinical settings, radiologists play an important role for assessing lesion resectability, evaluating recurrence, monitoring the biological behavior if an expectant management is chosen, and assessing response to systemic treatment as well as to radiation therapy.

Awareness of common locations, risk factors, and imaging features is fundamental for a correct diagnosis and an adequate patient management.

## Key points

- Illustrate typical locations, risk factors, and histology of DTs.
- Describe common imaging appearances of DTs.
- Review the principal differential diagnosis of abdominal DTs.

## Introduction

### Incidence, biological behavior, and classification

Desmoid tumors (DTs), also called deep/aggressive fibromatosis or desmoid-type fibromatosis, are rare (2/4 new cases per million people) and locally aggressive fibroblastic neoplasm [1, 2].

DTs frequently affect individuals between the ages of 15 and 60 years [3]. The accurate physiopathology remains unclear.

Further risk factors are prior trauma/surgery, pregnancy, and oral contraceptives [1]. The hormonal influence could explain why there is a female predilection (F:M = 2:1) and why DTs are more aggressive in younger patients [4].

**Fig. 1:**
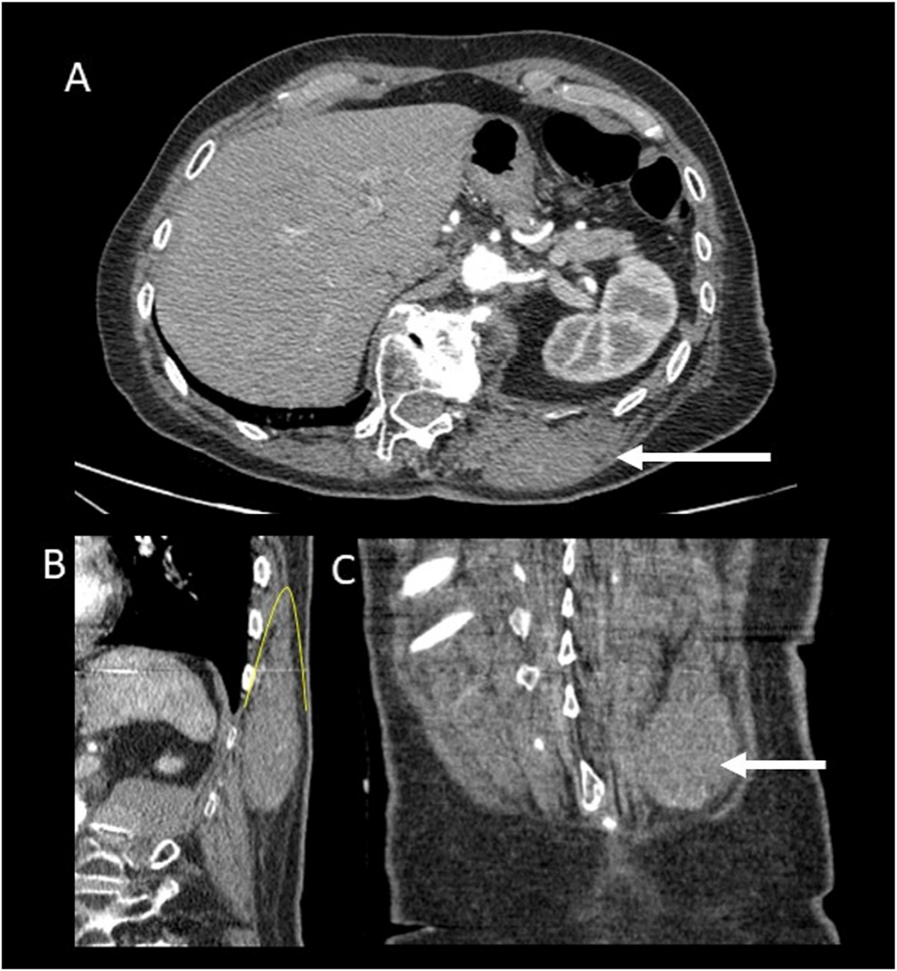
Patient with a previous history of non-Hodgkin lymphoma. CT imaging shows a homogenous mass located superficially to the left paravertebral muscle (arrow). In the oblique (**b**) and coronal (**c**), the fascial tail sign is shown (yellow line in **b**) indicating thin linear extension along the fascial plane. Histologic analysis confirms the diagnosis of paravertebral DT

DTs’ biological behavior is strongly heterogeneous: from an indolent behavior with also spontaneous regression (the so-called biologic burn-out) to a very locally aggressive tumor with a high rate of local invasion and recurrence due to the difficulty to achieve negative margins (Fig. 1).

Despite its high recurrence rate (20–68% especially within the first 1.5–5 years after treatment), it usually exhibits no distant metastatic potential [5–7]; otherwise, some anedottical cases are described [8]. It can be also multiple [9].

DTs can arise anywhere [1], so they are usually classified as follows:

- Extra-abdominal DTs
- Intra-abdominal DTs
- Abdominal wall DTs

The most common DTs are sporadic and extra-abdominal ones (extremities, head-neck, and chest wall/breast). The typical clinical presentation is a slow-growing painless or minimally symptomatic soft tissue mass.

Approximately 30% of patients have tumors related to familial adenomatous polyposis (FAP), and in these patients, intra-abdominal location is the most common type and can be also multifocal [10].

Intra-abdominal DTs’ clinical presentation can be typically as a slow-growing mass but can have also acute presentation such as intestinal ischemia or obstruction [11].

Otherwise, the most common location for pregnancy-associated DTs is the abdominal wall [12].

Knowledge of pathological appearance of DT can help to better understand also the radiological features of DTs and make easier for the radiologists to suggest the diagnosis of DTs [12, 13].

## Pathology features

The pathology features are the same in abdominal, intra-abdominal, and extra-abdominal desmoids.

Macroscopically, DTs are confined to the musculature and overlying aponeurosis or fascia and rarely (very large lesions) can infiltrate the subcutaneous tissues. They look like scar tissue without a true capsule. This infiltrative aspect is often misdiagnosed by radiologists due to DT well-circumscribed appearance on imaging [14].

Microscopically, DTs are poorly circumscribed and infiltrative. DTs are composed of dense collagenous stroma and long fascicles of band, uniform fibroblasts with low cellularity [3]. Pleomorphism and necrosis are not seen [14]. Mitoses are rare (up to 5 per 10 high power fields) [14].

At the periphery of the tumor, there are entrapped remnants of striated muscle that can go to atrophy that may be mistaken, also by radiologists, for evidence of malignant disease.

For all these reasons, the main differential diagnoses are low-grade fibrosarcoma and reactive fibrosis.

Immunohistochemistry plays an important role to confirm the diagnosis (such as positivity for smooth-muscle actin and for b1-catenin). Otherwise, nuclear immunoreactivity for b1-catenin is not pathognomonic due to the possibility of false-positive cases (such as superficial fibromatosis and low-grade myofibroblastic sarcomas) and false-negative cases [15–17].

The histopathologic confirmation is mandatory especially to rule out malignant tumor such as fibrosarcoma. A diagnosis of DT can be performed on core biopsies using 14G or 16G needles at a dedicated diagnostic clinic by a specialist radiologist in conjunction with a sarcoma surgeon. Neither incisional nor excisional biopsy is recommended as the initial diagnostic modality [18].

The biopsy has to be planned in such a way that the biopsy tract can be safely removed at the time of definitive surgery to reduce the risk of seeding [18].

Due to the rarity of DTs, misdiagnosed cases are about 30–40% during initial work-up also in reference centers [16, 19]. Pathology diagnosis requires a pathologist having expertise of musculoskeletal tumors [20].

US (ultrasound), CT (computed tomography), and MRI (magnetic resonance imaging) have different roles in the diagnosis of DT depending on its locations and clinical presentation.

The purpose of this article is to provide a comprehensive review of DT imaging appearance and its pathogenesis (Table 1). We will describe the DT multimodality imaging features, discuss possible alternative differential diagnosis, and review the role of imaging in surgical and conservative management.

**Table 1:** Adapted from [21]

**Epidemiology**	• ~ 0.03% of all neoplasms; < 3% of all soft tissue tumors. • 30% of *familial adenomatous polyposis* (FAP) patients have desmoid-type fibromatosis (also called *Gardner syndrome*); 7.5–16% of patients with fibromatosis have FAP. • Mean age: 36–42 years. • Female predominance from puberty to age 40; younger and older patients have M:F = ~ 1:1. • Male predominance in FAP of 3:1.
**Sites**	• 37–50% occur in the abdominal region. • Shoulder girdle, chest wall, and inguinal regions are the most prevalent extra-abdominal sites. o In FAP: majority (51–67%) are intra-abdominal or in the abdominal wall. o Sporadic: extra-abdominal are more common. • Abdominal wall is the prevalent site in premenopausal and pregnant women.
**Pathophysiology/etiology**	• CTNNB1 and APC gene mutations (up to 89% of cases). • High estrogen states and positive trauma history can lead to activation of the Wnt/β-catenin pathway.
**Prognostic factors**	• Local recurrence in 20–30%. • Margin status critical for local recurrence in primary tumors but not significant in recurrent presentations. • CTNNB1 S45F mutation associated with significantly increased risk of recurrence.

## Extra-abdominal DT

**Fig. 2:**
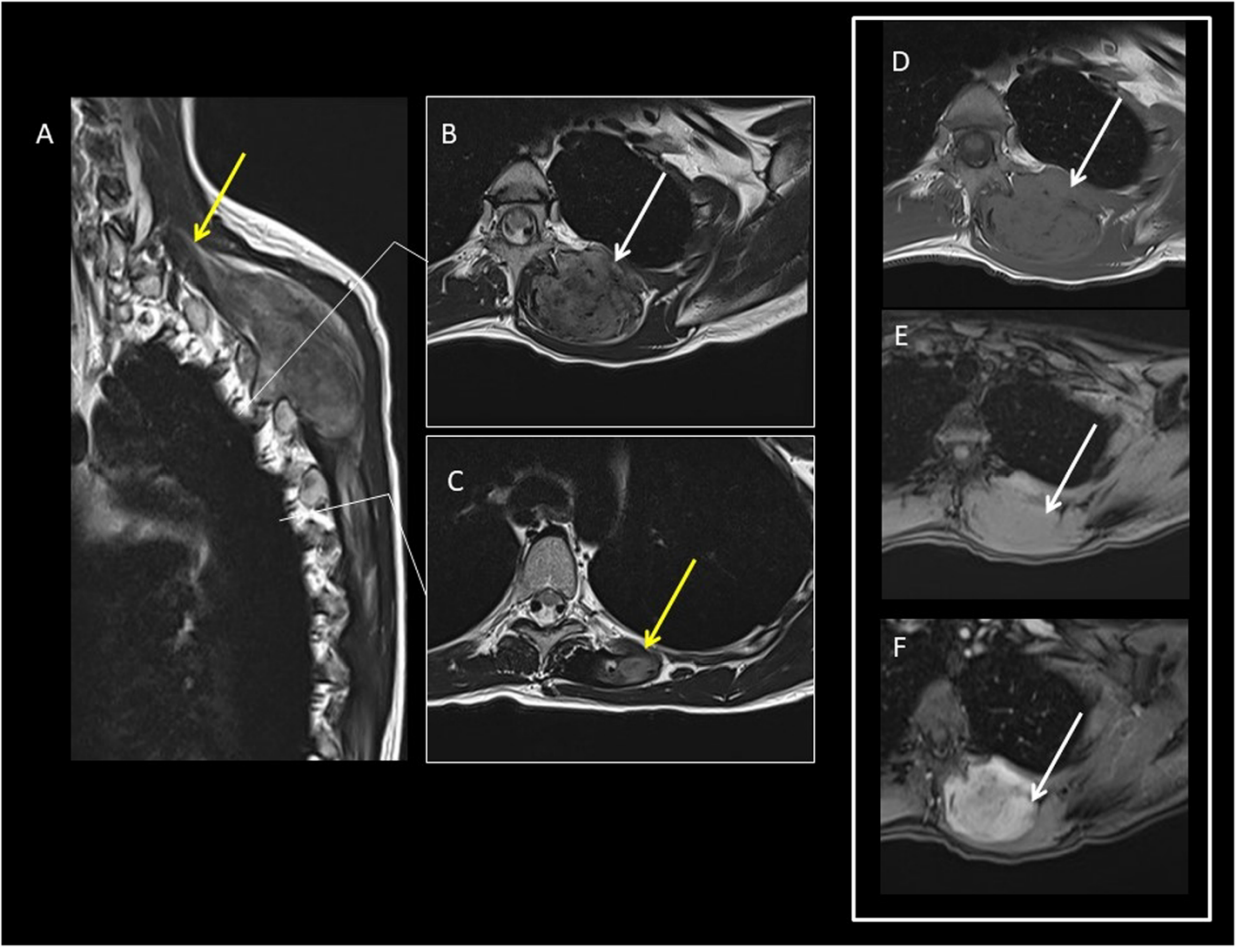
A 24-year-old female patient with DT located superficially into the left paravertebral muscle with infiltration of intercostal space. MR T2wi (sagittal (**a**) and axial plane (**b**, **c**)) showed a soft tissue mass with a heterogeneous hyperintense signal with band-like low-signal intensity (“band sign”). The lesion was isodense to the adjacent muscle on T1 (**d**) and fat sat T1 (**e**) and showed homogenous and late contrast enhancement. Especially, the sagittal plane (**a**) shows lesion extension along the fascial plane (yellow arrow, also in **c**)

**Fig. 3:**
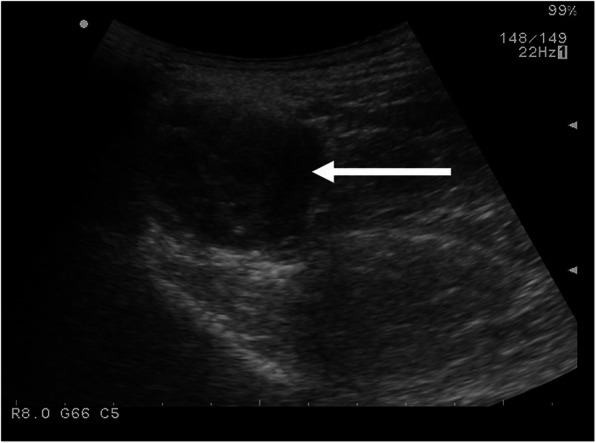
US appearance of a DT of the gluteal region in a 33-year-old female patient

**Fig. 4:**
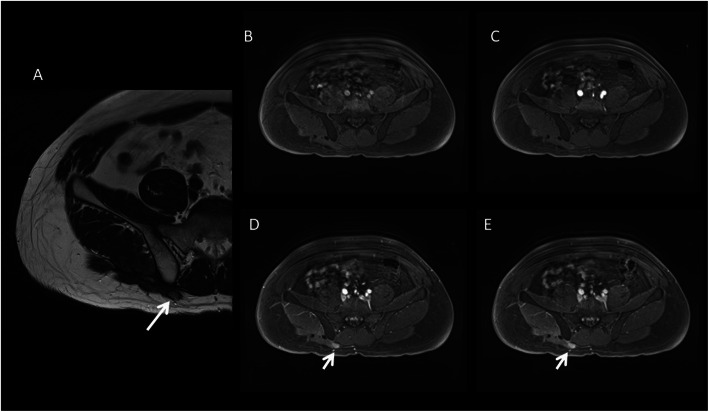
(Same patient of Fig. 3) After surgery, the MRI showed a small nodule (white arrow) characterized by mild hyperintensity on T2wi (**a**) and by late-progressive enhancement on dynamic T1wi (**b** T1 before contrast, **c** arterial phase, **d** portal phase, **e** late venous phase)

**Fig. 5:**
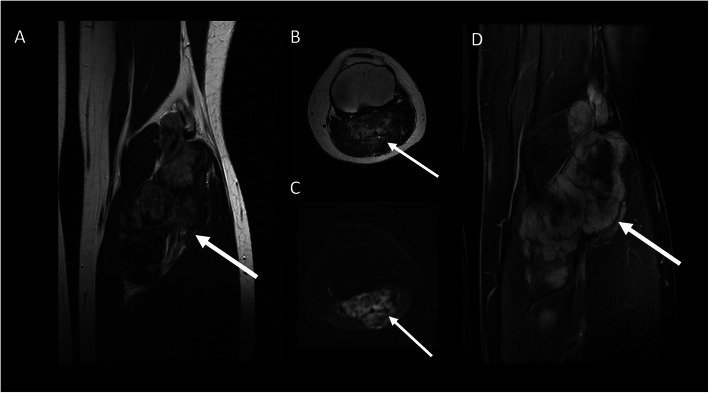
A 24-year-old female patient with DT of the popliteal fossa (arrow). T2-wi (coronal (**a**) and axial (**b**) plane) showed a soft tissue mass with a heterogeneous hyperintense signal with internal whirling or band-like low-signal intensity. The lesion is characterized by mild restriction on diffusion weighted images sequences (**c**). After contrast enhancement (T1 in **d**), the lesion was characterized by a peripheral contrast enhancement

The most common locations of extra-abdominal DTs are as follows [22–25]:

- 10% head and neck
- 33% shoulder and upper extremity
- 17% chest wall or back (Figs. 1 and 2)
- 30% gluteal region (Figs. 3 and 4) and lower extremity (Fig. 5)

DTs are multiple in 15% of cases [22].

Clinical presentation of extra-abdominal DT is typically a painless, deep soft tissue mass.

Ultrasound examination is the first-line imaging technique to evaluate a palpable mass.

DT is visualized as an oval, solid soft tissue mass with smooth or poor margins and variable echogenicity. Due to its heterogeneous composition, it can be characterized by alternate layers of hypo- (matrix and collagen) and hyperechogenicity (cells). Vascularization, evaluated at color Doppler US, can be variable.

An example of US features of a gluteal DT is shown in Fig. 3. It was surgically treated with recurrence as shown in Fig. 4.

Some radiological signs that can help radiologist to diagnose a DT are as follows:

- “Staghorn sign” due to intramuscular finger-like extensions [26].

This represents the local invasion (from the deeper tumor) along fibrous septa into the subcutaneous fat that resembles a branching stag-horn also at imaging [26].

- “Fascial tail sign” (“tail sign”) that corresponds to linear extension along fascial planes [22]. As dural tail sign for meningioma, it is described as a thin beak or linear extension along the orientation of the involved muscle fibers/aponeurosis (Figs. 1 and 2). This sign is useful especially in extra-abdominal DTs [27–29]. However, this is not pathognomonic of DTs and it is described also in soft tissue sarcomas that may arise in the fascia, especially in myxofibrosarcoma and malignant fibrous histiocytoma [30].

DTs show a MR heterogeneous appearance with variable signal on T2-weighted images (from iso- to hyperintense to skeletal muscle) and isointense signal on T1-weighted images [22] (Fig. 2).

The different intralesional components influence the MR signal intensity in the various imaging sequences as shown in Table 2 [3, 26]. Decreased T2 signal correlates to dense collagen and hypocellularity while increased T2 signal correlates with high cellularity.

**Table 2:** Relationship between MR signal intensity and histologic components [3]

DTs histologic components	MR signal intensity		
	T1wi	T2wi	Contrast enhancement
**Myxoid matrix**	Low	High	Intense
**Cellular stroma**	Intermediate to low	Intermediate to high	Moderate
**Fibrous tissue/collagen bands**	Low	Low	Absent

The so-called band sign is due to heterogeneous, band-like low-signal intensity on both T1- and T2-weighted images. It is considered as a distinguishing feature of DT and corresponds to the dense collagenous stroma. But this sign (useful also for abdominal wall and intra-abdominal DTs) is not pathognomonic because it may be visualized also in other benign and malignant soft tissue tumors (such as giant cell tumor of tendon sheath and myxofibrosarcoma).

Few studies have reported the features of the DTs on DWI: Oka et al. [31] found a significant difference in the ADC values between the DTs and malignant soft tissue tumors.

The higher ADC value of the DTs seems to be due to low cellularity and fibrous content [31]. This information might be promising, but more studies with larger patient series are required.

The contrast enhancement is variable too: 90% of the lesions demonstrate moderate-to-marked enhancement, especially in the more cellular [32]. Non-enhancing areas due to necrosis are very rare.

Extra-abdominal DTs typically have an intermuscular location along the deep fascia, and other important radiological signs at MRI are as follows [3]:

- *Split fat sign* that corresponds to a thin rim of surrounding fat
- *Flame signs*, feathery margins resembling a flame.

**Fig. 6:**
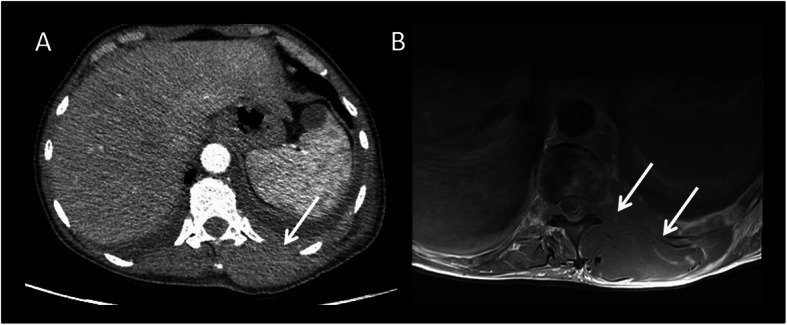
Patient affected by non-Hodgkin lymphoma. **a** CT imaging shows a lesion that infiltrates the left paravertebral muscle with extension in the paravertebral space. **b** MRI confirms an infiltrative mass with substitution of the left paravertebral muscle, the left adipose paravertebral space, and the posterior costal arch. This type of growth and behavior is strongly suggestive for a localization of lymphomatous disease

As mentioned before, despite all these typical imaging features, biopsy is still necessary to confirm the imaging impression and to distinguish DTs from other soft tissue tumors (Fig. 6).

## Intra-abdominal DT

Abdominal DTs have a typical higher incidence in female than in male patients, and this data is more evident than in the other locations [32–34].

Otherwise, the relationship between estrogenic stimulus and desmoid formation is not completely understood: some authors described estrogen receptor expression in tumor tissue and clinical efficacy of the antiestrogen therapy. But in many other case series, the lack of female predilection revealed that elevated estrogen levels are not essential for the development of DTs [34].

Also, positive history for previous trauma or surgery is considered as a possible risk factor (almost 75% of DTs have a positive previous history of abdominal surgery) [35].

Intra-abdominal DTs’ incidence significantly differs between sporadic and FAP-related cases: only 5% of sporadic DTs are intra-abdominal ones, whereas 80% of cases of FAP-related DTs are intra-abdominal (especially mesenteric ones) [36–38].

Abdominal DTs can be divided into pelvic or mesenteric lesions.

### Mesenteric DT

**Fig. 7:**
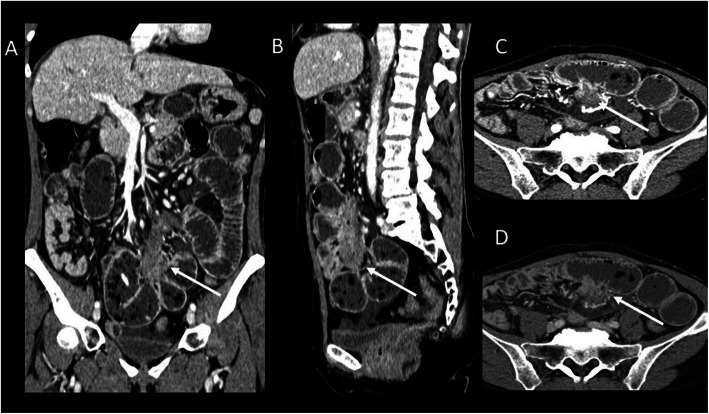
Mesenteric DT. A 54-year-old female patient with a positive history for previous surgery (hystero-annessiectomy) and chronic liver disease. CT images ((**a**) coronal, (**b**) sagittal, and (**c**, **d**) axial plane) show a poorly defined border lesion (arrow) with radiating spicules extending into the adjacent mesenteric fat and infiltrating the small bowel causing small bowel obstruction. Axial arterial phase (**c**) and portal phase (**d**) show progressive contrast enhancement of the lesion

**Fig. 8:**
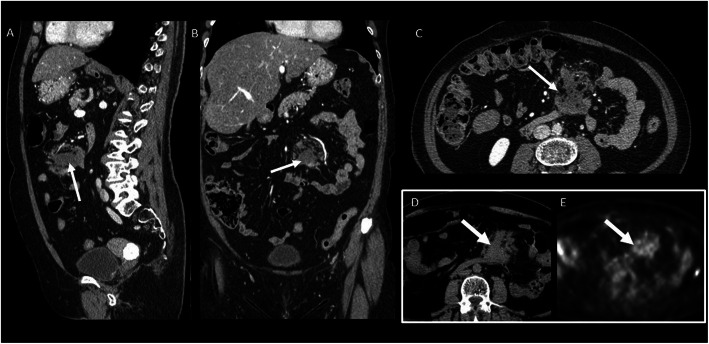
Asymptomatic patient performed a CT in order to stage a bladder cancer. Contrast-enhanced CT images ((**a**) sagittal, (**b**) coronal, and (**c**) axial planes) incidentally visualized ill-defined mass in the mesentery characterized by a mild contrast enhancement (white arrow). At CT-PET examination, the mass shows a mild to moderate uptake

**Fig. 9:**
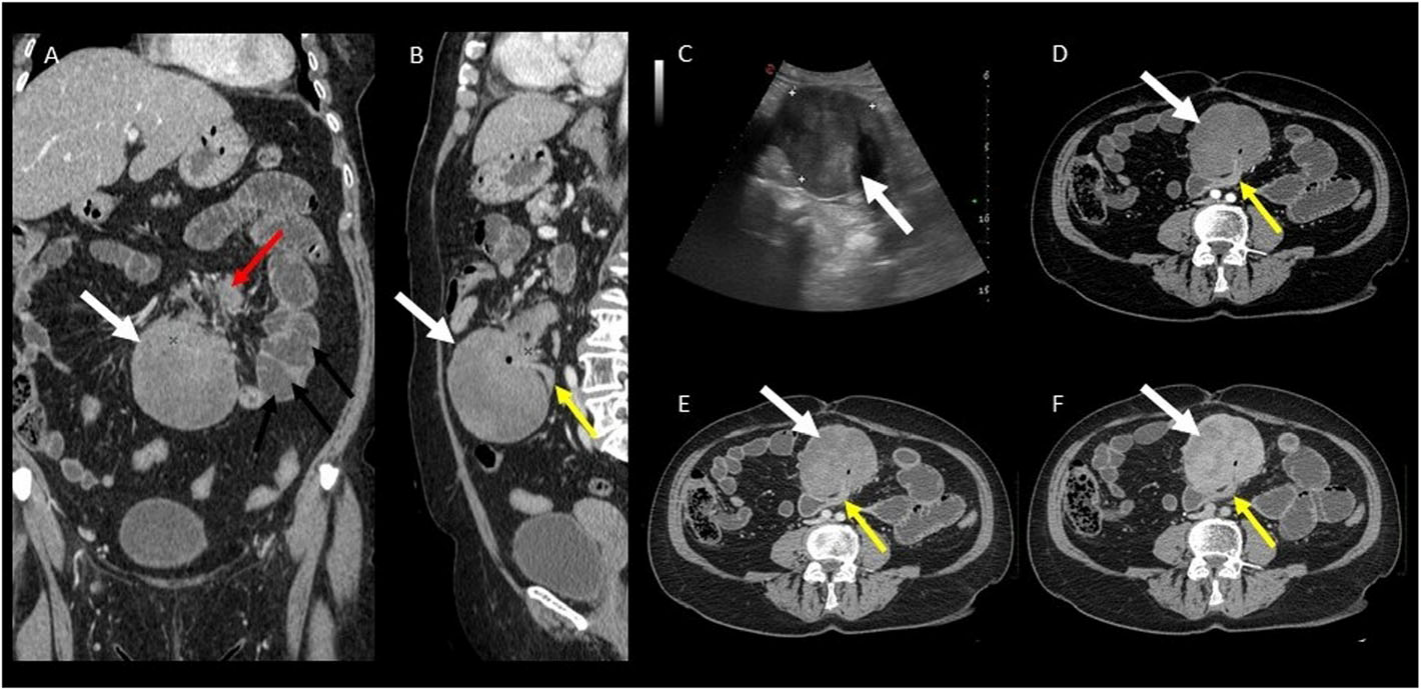
(Same patient of the previous figure) After 3 years of negative follow-up of the mesenteric mass, the patient was admitted to the emergency room with abdominal pain. CT examination ((**a**) coronal and (**b**) sagittal planes) shows a significant growth of mesenteric mass. In particular, it is possible to appreciate two different components: the ill-defined mass previously described (red arrow) and a new large, well-defined mass (white arrow). After contrast administration, the mass is characterized by progressive and homogeneous contrast enhancement ((**d**) arterial, (**e**) portal and late venous phase). Moreover, the lesion infiltrates/tethers duodenal loop (yellow arrow) causing overdistension of small bowel (black arrows). US examination (**c**) shows a well-defined homogenous mesenteric mass. Histologic findings confirmed the diagnosis of mesenteric DT

Mesenteric DT is the most common primary tumor of the mesentery (Figs. 7, 8, and 9).

The clinical presentations range from a painless palpable abdominal mass to bowel obstruction or perforation and chronic hydronephrosis due to ureteral infiltration.

Mesentery is the most common DT location in the Gardner syndrome [36].

CT is considered as the first level imaging technique for a prompt diagnosis of intra-abdominal DT and their possible acute complications especially in the Emergency Department.

CT scans show mesenteric DT often appearing as soft tissue mass with ill-defined margins and radiating spicules extending into the adjacent mesenteric fat (“whorled appearance,” Figs. 7 and 8), but it can also appear as a well-demarcated lesion (Fig. 9) [36, 37].

DTs are usually visualized as a large mass (> 15 cm), isodense to the muscle. Uncommonly, it can entrap ureters or encase small bowel loops, leading to intestinal perforation or obstruction [39]. Otherwise, in patients with FAP, lesions are smaller and multiple [35].

MR imaging is considered as a second level imaging technique especially for preoperative evaluation of mesenteric masses in selected cases.

CT and MR imaging DT features depend on histological and vascular characteristics (see Table 2).

At PET-CT examination, DTs’ uptake pattern of fluorodeoxyglucose ranges from low to moderate grade. A potential clinical role of PET-CT (especially in FAP patients) is to differentiate DTs (low to mild uptake) from recurrent cancer (moderate to high uptake) [3] (Fig. 8e).

Mesenteric DTs have to be distinguished from other mesenteric lesions (primary and metastatic ones).

**Fig. 10:**
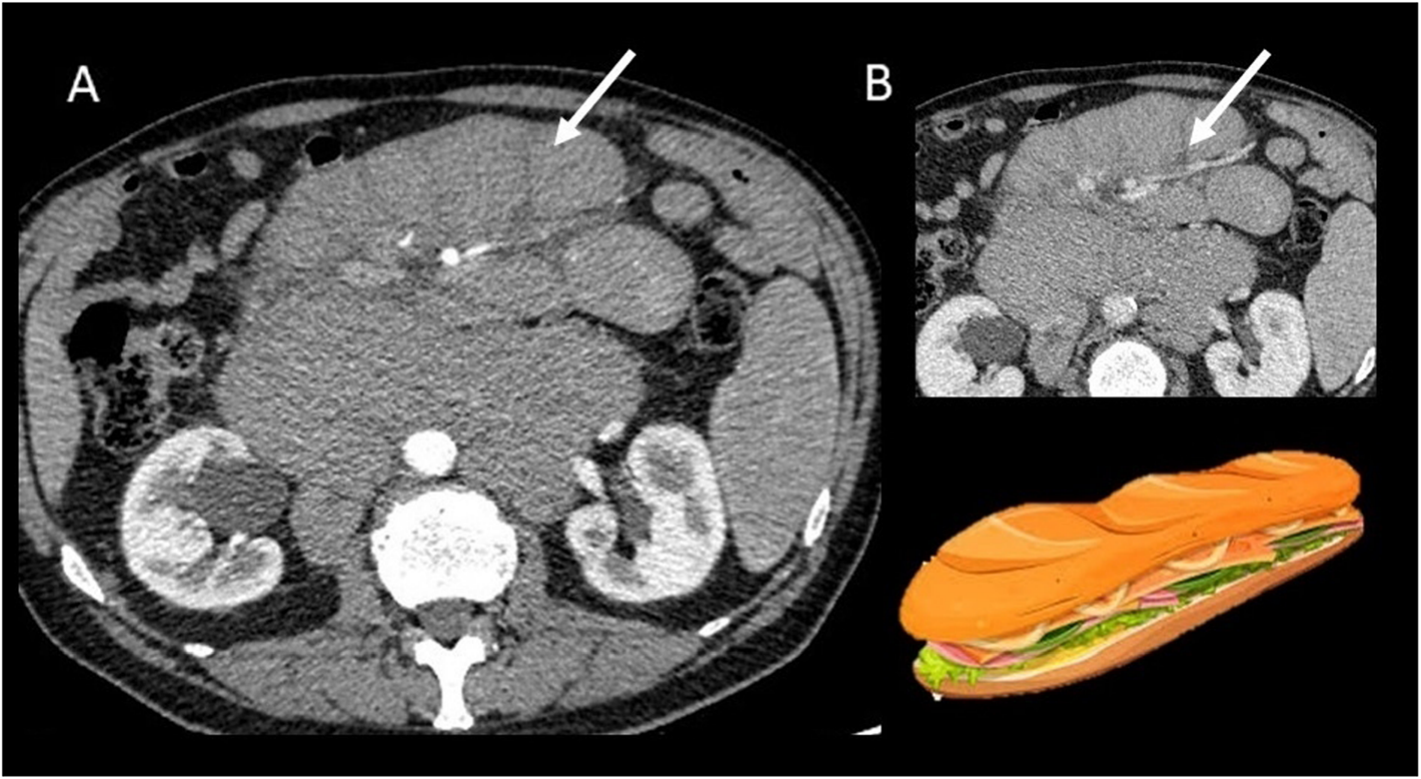
Patient affected by non-Hodgkin lymphoma with both retroperitoneal and mesenteric locations. CT images show a large lesion with well-defined margins and homogeneous enhancement both on arterial (**a**) and portal (**b**) phases. Confluent lymphadenopathy on both sides of the mesenteric vessels gives rise to an appearance described as the sandwich sign that is specific for mesenteric lymphoma

Mesenteric lymphomas typically manifest as a large lesion with well-defined margins, homogeneous attenuation that encases mesenteric vessels (“sandwich sign”), and involve the adjacent small bowel segments (Fig. 10) [40].

**Fig. 11:**
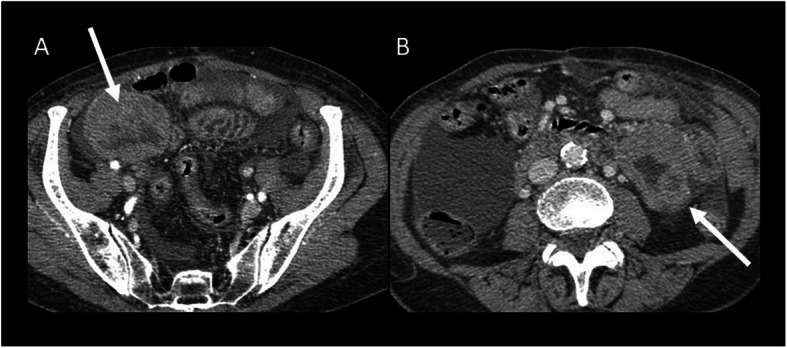
Ileal and digiunal GIST. CT shows two masses (arrow) with an extensive mesenteric component characterized by central necrosis, which manifest as focal areas of hypoattenuation

Gastrointestinal stromal tumors (GISTs) can infiltrate the mesentery (especially in case of large small bowel GISTs with an extensive mesenteric component) or can originate primarily in the mesentery [39]. Intralesional hemorrhage and central necrosis (visualized as a central area of hypoattenuation) are typical features of GISTs (Fig. 11).

**Fig. 12:**
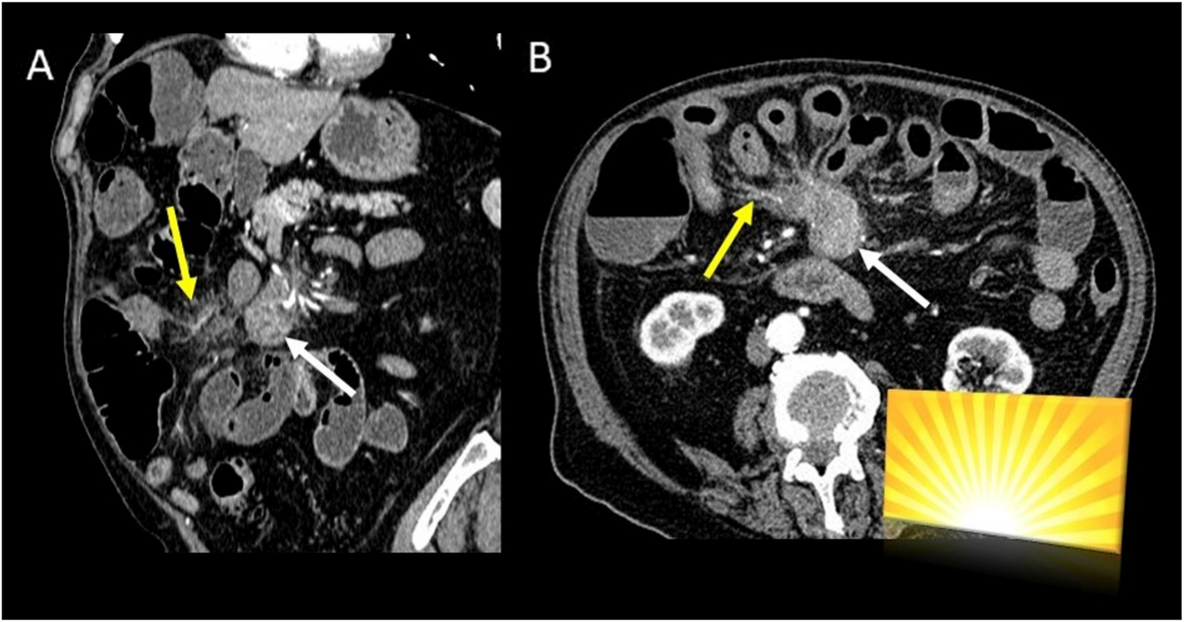
Mesenteric carcinoid tumor. CT images ((**a**) coronal and (**b**) axial plane) show hypervascular mesenteric mass (white arrow) with intralesional calcification and an intense fibrotic proliferation (yellow arrow), the so-called spoke-wheel or sunburst sign

Metastatic carcinoid tumor and sclerosing mesenteritis can simulate a primary mesenteric neoplasm at CT scan (Fig. 12).

Namely, the primary intestinal carcinoid tumor is often occult at CT (due to its small dimension) explaining why the mesenteric mass is usually the dominant imaging finding. CT examinations usually show an hypervascular mesenteric mass associated to intralesional calcification (up to 70%) and to an intense fibrotic proliferation and desmoplastic reaction in the mesenteric fat due to the release of serotonin by the primary tumor [41], the so-called spoke-wheel or sunburst sign (Fig. 12).

In adults, mesenteric soft tissue sarcomas (such as liposarcoma) have to be considered as possible differential diagnosis.

### Pelvic DT

**Fig. 13:**
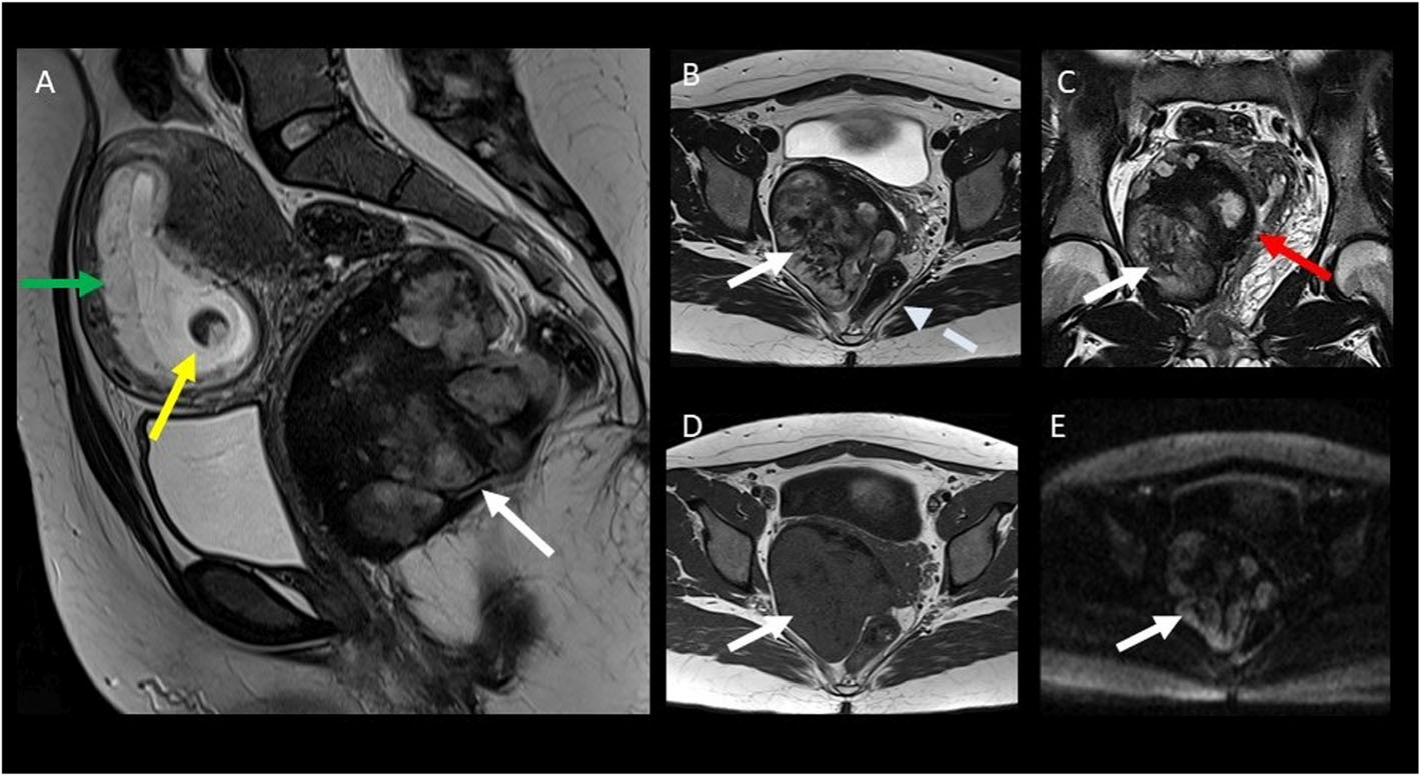
A 22-year-old female patient with pelvic DT onset during pregnancy (white arrow). T2wi (sagittal (**a**), axial (**b**), and coronal (**c**) plane) showed a soft tissue well-defined mass with a heterogeneous hyperintense signal with internal band-like low-signal intensity. The lesion is characterized by mild restriction on diffusion-weighted image sequences (**e**) and isointensity signal to the muscle on T1wi (**d**). The lesion did not infiltrate the rectum (dot arrow in **b**) but was not cleavable from the lateral wall of the vagina (red arrow in **c**). Green arrow in **a** showed the anterior placenta, and yellow arrow in **a** showed the fetus head

Pelvic DTs are considered as a variant of abdominal desmoids due to their location in the iliac fossae and pelvis (Fig. 13).

Its typical presentation is asymptomatic slow-growing palpable mass. But larger lesions can infiltrate the urinary bladder, the vagina, or the rectum and can cause hydronephrosis. Moreover, pelvic DTs can compress the iliac vessels and may be clinically mistaken for an adnexal lesion. Although it occurs especially in young women, it is still not considered as a pregnancy-related tumor; otherwise, we propose a case of pelvic DT onset during pregnancy [42].

## Abdominal wall DT

Abdominal wall DT typically originates from musculo-aponeunotic structures of the abdominal wall and rectus, and internal oblique muscles are the most common site.

It is considered as the most frequent pregnancy-related desmoid tumor, and it can occur not only during pregnancy but also during the first year after childbirth [43]. Areas of previous abdominal surgery and cesarean scar can be the site of origin of DT.

As mentioned before, the role of hormonal stimulation is controversial also in pregnancy-related desmoid tumors. Significant estrogen receptor expression has been reported in very few desmoids. Some authors speculate that the “trauma” due to the stretching of the abdominal wall during pregnancy can be the trigger for DTs’ formation. But this hypothesis does not explain why DT (during and after pregnancy) can originate also in other sites and why some DTs have a spontaneous regression after pregnancy. Furthermore, it is reported that some DTs, both in pregnant and non-pregnant patients, respond to antiestrogen treatment [44].

**Fig. 14:**
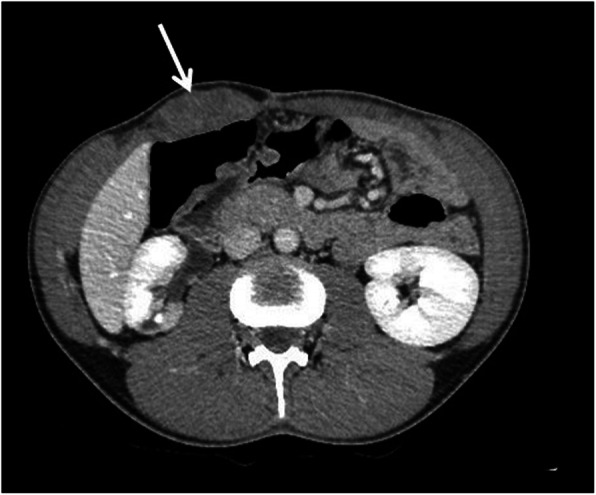
CT appearance of an abdominal wall DT (arrow) in a 26-year-old male patient affected by Gardner syndrome with previous total colectomy

Abdominal wall DTs can be also associated to FAP (Fig. 14).

DT development is not only an estrogen-related phenomenon but more complicated, and still unknown hormonal stimulations may be present.

Possible differential diagnoses at this location are abdominal wall endometriosis and hematomas of the abdominal wall.

**Fig. 15:**
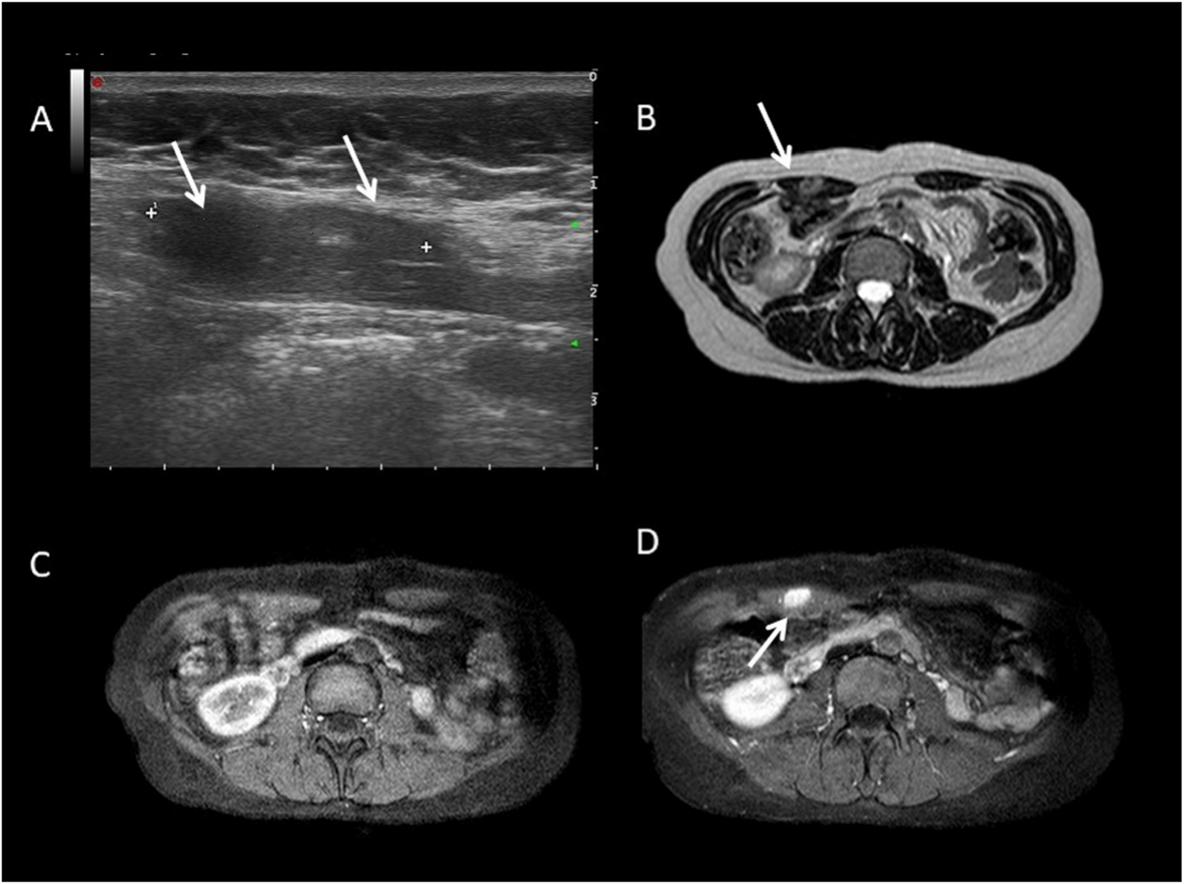
Young female patient with a positive history for pregnancy and cesarean section. Abdominal wall DT (white arrow) appears as homogeneously hypoechoic masses at US examination (**a**). On MRI, it is characterized by T2 hyperintensity on T2wi (**b**) and homogeneous enhancement on fat sat T1wi (**d**). Fat sat T1wi before contrast (**c**)

**Fig. 16:**
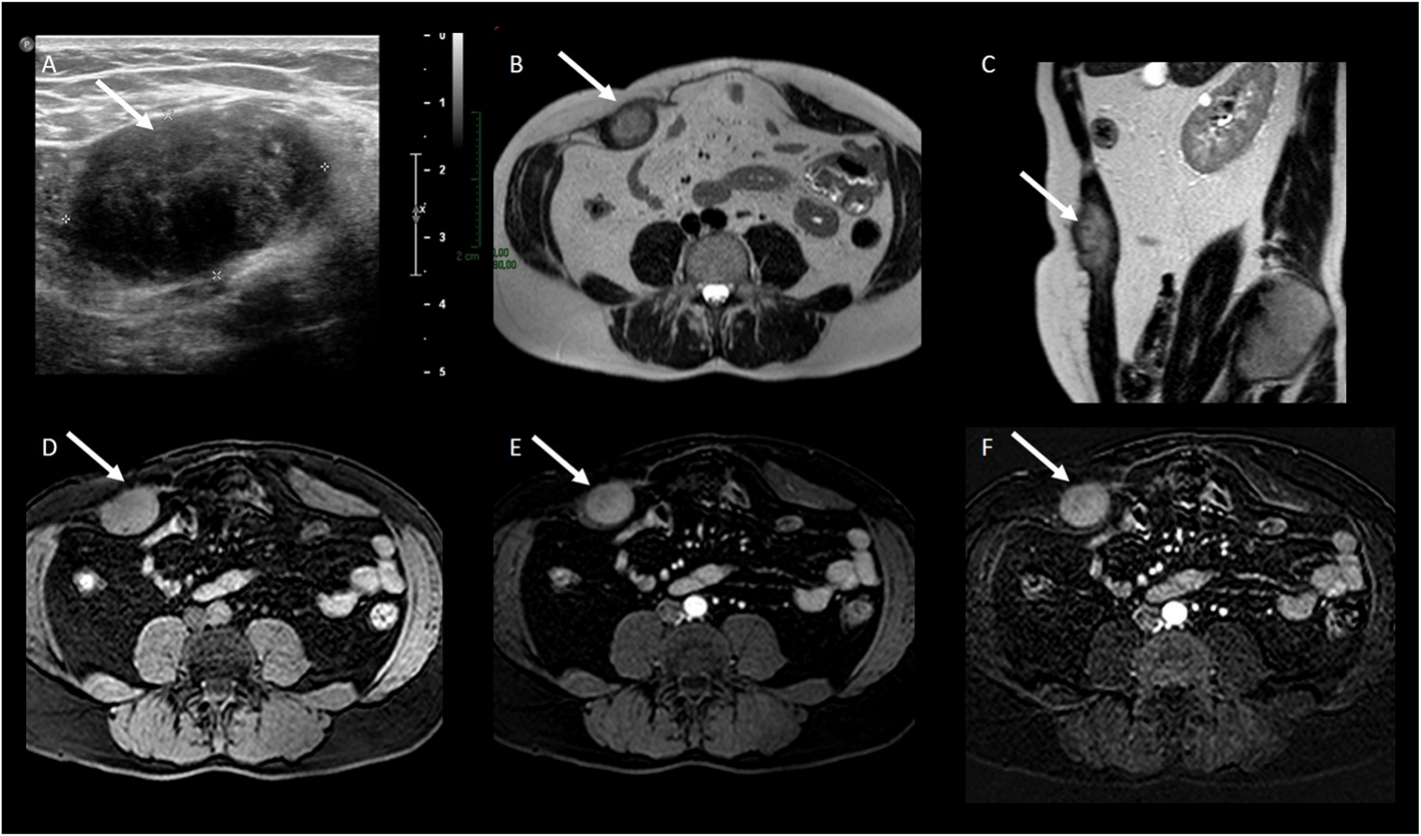
DT of the abdominal wall. **a** US examination showed a well-defined mass in the right rectus muscle. At MRI, the lesion is characterized by hyperintense signal on T2wi (**b**, **c**), isointense signal with the muscle on T1wi (**d**), and homogeneous enhancement on T1wi after contrast agent administration (**e**) especially on subtracted imaging (**f**)

US is particularly appropriated for diagnosis and follow-up of pregnancy-associated DT of the abdominal wall due to the lack of ionizing radiation. MRI can be considered as a second level technique in selected cases (DTs show the same features as in the other location) (Figs. 15 and 16).

Abdominal wall endometriosis affects young woman and could be associated to deep endometriosis. A positive history of previous surgery is considered one of the possible causes of “seeding” of endometrial cells.

At US examination, abdominal endometriosis appears as an oval, hypoechoic solid lesion in the subcutaneous fat, muscle, or fascial layers.

At MR, the lesion can be hyperintense on T1-weighted imaging due to the presence of blood products but some lesions may have an intermediate to low signal intensity on T1- and on T2-weighted imaging for a prominent fibrous component [45].

With the aging of population and the widespread use of anticoagulant medications, abdominal wall hematoma (especially rectus sheath hematoma) is a common finding especially in older patients. It shows the typical T1 hyperintensity from subacute blood products and contrast enhancement after contrast administration. MRI is often not necessary: correlation to the clinical history is often enough.

In the correct clinical scenario, CT examination before and after contrast administration can be used to evaluate active bleeding.

## Role of imaging in DT

The primary role of imaging examination is to define the extension and potential resectability of the lesion.

Presently, guidelines recommend observation (“wait-and-see policy” or “expectant management”) as a primary treatment option for unresectable tumors or resectable but asymptomatic tumors [46].

If DTs are managed non-operatively, periodic imaging assessment is mandatory, especially if intra-abdominal (3–6-month interval). CT is applied to monitor intra-abdominal DTs, and MR imaging is the modality of choice for the follow-up of extra-abdominal and pelvic DTs [46, 47].

**Fig. 17:**
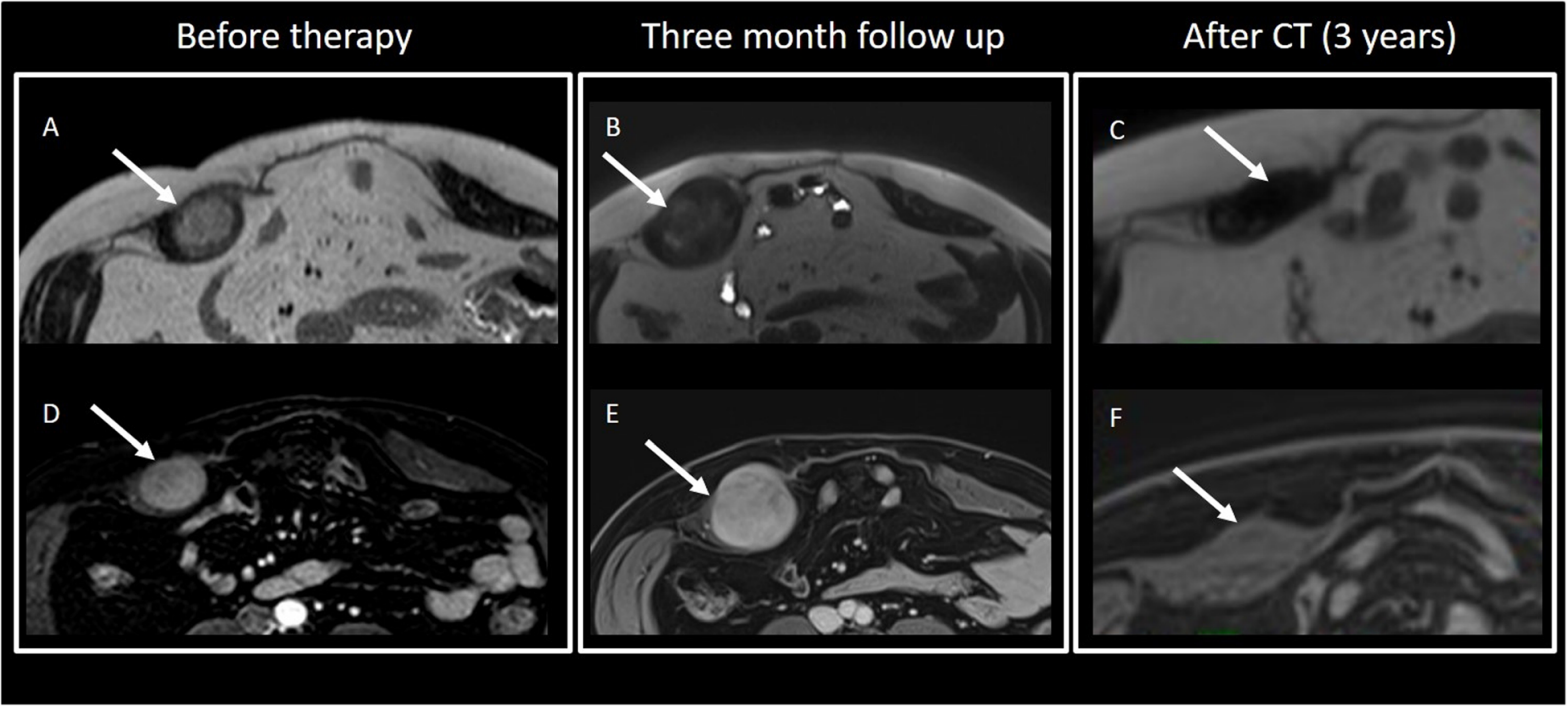
(Same patient of Fig. 16) Abdominal wall DT (T2wi in **a** and after contrast T1wi in **b**). Patient underwent to “wait and see approach” and then to CT therapy. MRI showed the variation of the signal on T2wi and of the contrast enhancement of the lesion (white arrow): after 3 months of follow-up, the lesion increased in dimension, maintained moderate contrast enhancement on T1wi (**e**), but became less hyperintense and more disomogeneus on T2wi. Then, the patient started a CT protocol with methotrexate and vinorelbine. After 3 years, the lesion significantly reduced its size, become strongly hypointense on T2wi (**c**), and did not show any significant contrast enhancement (**f**)

**Fig. 18:**
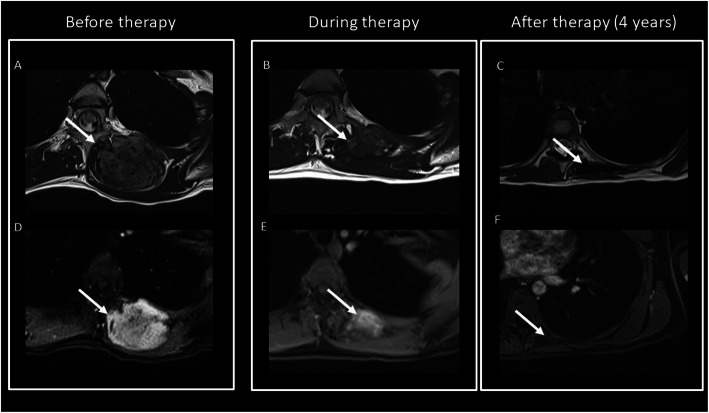
Chest posterior wall DT (T2wi (**a**) and after contrast T1wi (**d**)). A 23-year-old female patient underwent chemotherapy. MRI showed progressive decrease of T2wi signal intensity and contrast enhancement on T1wi of the lesion (white arrow): during therapy, the lesion decreased in dimension and in T2 hyperintensity (**b**) but maintained moderate contrast enhancement on T1wi (**e**). After 4 years, the lesion significantly reduced its dimension, become hypointense on T2wi (**c**), and did not show significant contrast enhancement (**f**)

MRI is preferred due to the prognostic value of the T2 signal and enhancement (Figs. 17 and 18): higher T2 signal and contrast enhanced seem to be associated to a more rapid growth rate (Table 3) [48].

**Table 3:** MR imaging signal intensity and phases of DTs [3]

MR signal intensity	Growth phase	Plateau phase	Regression phase
**T1-weighted**	Low	Low	Very low
**T2-weighted**	High	Low	Very low
**Enhancement**	Mild to moderate	Mild	Absent

Due to the potential risk of recurrence, imaging follow-up has been recommended after therapy, initially every 3–6 months [46, 47].

After chemoradiation, imaging intervals have to be regulated on the basis of the growth rate and presence of symptoms.

## Conclusion

DTs are uncommon, locally aggressive tumors with a high risk of recurrence. There are several therapeutic options, including “wait-and-see policy,” surgery, radiotherapy, chemotherapy, and hormonal and molecular targeted drugs. A multidisciplinary approach for a “tailored therapy” is usually needed: in this clinical scenario, radiologists play a crucial role to make a correct diagnosis and to guide the proper management, depending on the location, imaging features, and clinical presentation of these kinds of masses.

## Abbreviations

CT: Computed tomography
DTs: Desmoid tumors
FAP: Familial adenomatous polyposis
MRI: Magnetic resonance imaging
US: Ultrasound
